# MiR-760 suppresses human colorectal cancer growth by targeting BATF3/AP-1/cyclinD1 signaling

**DOI:** 10.1186/s13046-018-0757-8

**Published:** 2018-04-16

**Authors:** Ling Cao, Yulin Liu, Dan Wang, Lan Huang, Feng Li, Jinbo Liu, Chaoqi Zhang, Zhibo Shen, Qun Gao, Weitang Yuan, Yi Zhang

**Affiliations:** 1 Biotherapy Center, The First Affiliated Hospital of Zhengzhou University, Zhengzhou, 450052 Henan China; 2 Cancer Center, The First Affiliated Hospital of Zhengzhou University, Zhengzhou, 450052 Henan China; 3 School of Life Sciences, Zhengzhou University, Zhengzhou, Henan 450001 People’s Republic of China; 4 Henan Key Laboratory for Tumor Immunology and Biotherapy, Zhengzhou, 450052 Henan China; 5 Department of Colorectal Surgery, The First Affiliated Hospital of Zhengzhou University, Zhengzhou, 450052 Henan China

**Keywords:** microRNA-760 (miR-760), Colorectal cancer (CRC), Basic leucine zipper transcriptional factor ATF-like 3 (BATF3), Activator protein 1 (AP-1)

## Abstract

**Background:**

Recent studies have reported that microRNAs (miRNAs) often function as negative post-transcriptional regulators with altered expression levels found in colorectal cancer (CRC). There have been few studies on miRNAs that regulate the oncogenic alterations in CRC. Here, we aim to explore the anti-cancer miRNA and the potential mechanisms by which miRNAs modulate CRC progression.

**Methods:**

We performed an integrated analysis of CRC miRNA expression datasets in The Cancer Genome Atlas (TCGA). The miRNA with the lowest expression, miR-760, was validated in an independent validation sample cohort of 76 CRC tissues. Functional assays, such as CCK-8 assay, colony formation assay, and CFSE staining, were used to determine the oncogenic role of miR-760 in human CRC progression. Furthermore, western blotting and dual-luciferase reporter assay were used to determine the mechanism by which miR-760 promotes proliferation of CRC cells. Xenograft nude mouse models were used to determine the role of miR-760 in CRC tumorigenicity in vivo. Immunohistochemical assays were conducted to study the relationship between miR-760 expression and basic leucine zipper transcriptional factor ATF-like 3 (BATF3) expression in human CRC samples.

**Results:**

miR-760 was markedly downregulated in CRC tissues, and low miR-760 expression was associated with poor prognosis among CRC patients. Upregulation of miR-760 suppressed CRC cell proliferation, whereas downregulation of miR-760 promoted CRC proliferation in vitro. Additionally, we identified BATF3 as a direct target of miR-760, and that the essential biological function of miR-760 during CRC progression both in vitro and in vivo is to suppress the expression of BATF3 and downstream cyclinD1 via AP-1 transcription factor. Finally, we showed a significant correlation between miR-760 and BATF3 expression in CRC tissues.

**Conclusions:**

miR-760 inhibited CRC growth by downregulating BATF3/AP-1/ cyclinD1 signaling.

**Electronic supplementary material:**

The online version of this article (10.1186/s13046-018-0757-8) contains supplementary material, which is available to authorized users.

## Background

Colorectal cancer (CRC) is one of the most common malignancies in the world, with high rates of incidence and disease-related mortality and morbidity [1]. Approximately 5-10% of colorectal cancers are caused by heritable mutations. Scientists think that roughly 50% of the remaining cases are caused by sporadic mutations [2]. However, the precise mechanisms involved in CRC tumorigenesis are still not well understood. MicroRNAs (miRNAs) are small noncoding RNA molecules that modulate the expression of target mRNAs at a post-transcriptional level [3]. They regulate critical aspects of the oncogenic phenotype through the disruption of protein translation by selective binding and degradation of target mRNAs [4]. An increasing number of studies have shown that dysregulation of miRNA expression plays an important role in cancer initiation, progression, and prognosis, as well as acquired resistance toanticancer agents [5–7]. Several miRNAs have been found to participate in the pathogenesis of CRC, including miR-21, miR-451, miR-499-5p, miR-375, and miR-142-5p [8]. However, there have been few studies examining miRNAs that regulate the oncogenic mutations in CRC.

The AP-1 (activator protein 1) transcription factor is a dimeric complex that comprises members of the JUN, FOS, ATF (activating transcription factor), and MAF (musculoaponeurotic fibrosarcoma) protein families [9]. AP-1 factors regulate diverse cellular processes, including differentiation, proliferation, and cell survival, and are also critically involved in the development of various cancers [10–13]. They contain a basic DNA binding domain and a leucine zipper dimerization domain, and dimers preferentially bind to consensus TGA(C/G)TCA (TRE) or TGACGTCA (CRE) DNA motifs [14]. Several studies have reported that functional loss or downregulation of AP-1, or post-transcriptional control of JunD by miRNA can effectively suppress the proliferation of cancer cells [15, 16]. However, post-transcriptional regulation of AP-1 in CRC cells was rarely mentioned as in other cancers. Basic leucine zipper transcription factor ATF-like (BATF), BATF2, and BATF3 belong to the AP-1 family of transcription factors that regulate numerous cellular processes. Initially, BATF family members were thought to function only as inhibitors of AP-1-driven transcription [17–19]; however, recent studies have uncovered that these factors have unique, non-redundant, and positive transcriptional activities in dendritic cells [20], B cells, and T cells [21–23]. Given their functional redundancy [24], this suggests BATF3 might also has oncogenic potential.

In the present study, we performed an integrated analysis and identified miRNAs with altered expressionin CRC cells using miRNA sequencing data from The Cancer Genome Atlas (TCGA, http://tcga-data.nci.nih.gov/docs/publications/coadread_2012). We identified miR-760 as a clinically noteworthy miRNA in CRC and confirmed that miR-760 expression was lower in CRC patients with poor survival. We further found that ectopic expression of miR-760 suppresses proliferation and tumorigenicity in CRC cells. Moreover, our data indicated that the full-length 3′-untranslated region (3′-UTR) of human BATF3 mRNA was a direct target of miR-760. Thus, miR-760 plays an essential role during the regulation of BATF3 in CRC cells in vitro and in vivo. Our present study suggests that miR-760 suppresses cell proliferation and tumorigenicity in CRC cells by targeting BATF3 mRNA and suppressing the expression of BATF3 and downstream cyclin D1.

## Methods

### Cell line culture and morphological observation

The human CRC cell lines SW620, DLD1, and HCT116, and colorectal mucosa cell line FHC were purchased from the cell bank of the Chinese Academy of Sciences (Shanghai, China) and cultured in RPMI 1640 medium (Invitrogen Life Technologies, Carlsbad, CA, USA) supplemented with 10% fetal bovine serum (Gibco), 100 U/ml penicillin, and 50 mg/ml streptomycin. All cells were incubated at 37 °C with a humidified atmosphere of 5% CO_2_. The morphology of the CRC cells was observed using phasemicroscopy (Leica,Wetzlar, Germany).

### Patients, specimens, and follow-up

In this study, CRC samples used in the immunohistochemistry (IHC) assay were obtained randomly from patients who underwent radical resection of pathologically confirmed tumors between 2013 and 2015 in the First Affiliated Hospital of Zhengzhou University (Zhengzhou, Henan, China). None of the patients received any preoperative anticancer treatment. The present research was approved by the research ethics committee of the First Affiliated Hospital of Zhengzhou University. The group of patient population used in different experiments was listed in Additional file 1: Table S1. A total of 80 cases were used in this study to examine BATF3 expression, among which 45 samples were obtained from patients between 2014 and 2015 that tested positive for miR-760 expression, and the other 35 samples were obtained from patients in 2013 from the Department of Pathology at the First Affiliated Hospital of Zhengzhou University. All patients included were monitored until 2017, with a median observation time of approximately 33 months. All patients provided written informed consent to participate in this study.

### Vectors, lentiviral infection, and transfection of miR-760 mimic, inhibitor, and siRNA of target gene

The miR-760 gene was PCR-amplified from genomic DNA and cloned into a psicoR-GFP lentiviral vector. Psico-miR-760 was cotransfected with the psPAX2 and pMD2.G packaging plasmid in HEK293 T cells using Lipo3000 (Invitrogen Life Technologies, Carlsbad, CA, USA) following the manufacturer’s instructions. At 48 h after the cotransfection, supernatants were collected and incubated with CRC cells for a 24-h infectionperiod in the presence of polybrene (5 μg/ml). After infection, GFP-positive cells were sorted by MoFloXDP (Beckman, USA) to obtain miR-760 overexpression cells. The 3′-UTR of BATF3 was amplified and cloned downstream to the luciferase gene in a modified pmirGLO control vector (received as a gift from Professor Guoqiang Zhao, Zhengzhou University). Hsa-miR-760 mimic, the negative control (NC) mimic, hsa-miR-760 inhibitor, and the NC inhibitor were purchased from Genepharma Company (Shanghai, China).

### RNA extraction and quantitative reverse transcription PCR (qRT-PCR)

As described in our previous study, total miRNA from cultured cells and CRC tissues were extracted using Trizol solution (Invitrogen Life Technologies) [25]. Independently, RNA from each sample was reverse-transcribed using PrimeScript RT reagent Kit (Takara Bio, Otsu, Shiga, Japan). Subsequently, expression levels of miR-760 were quantified by qRT-PCR using SYBR Premix ExTaqII (TaKaRa, Japan) in Agilent Mx3005P. Expression levels of genes were normalized to that of the housekeeping gene GAPDH as the control. miRNA expression was defined based on Ct values, and relative expression levels were normalized according to the expression of small nuclear RNA U6. We used melting curves to monitor non-specific amplifications. The 2^-ΔΔCt^ method was used to calculate relative expression changes.

The following primers were used:

miR-760 forward, 5′-CGGCTCTGGGTCTGTGGGGA-3′;

BATF3 forward, 5′-AGAGAGATCGGGAAGCTGACA-3′;

P21 forward, 5′-GAGCGATGGAACTTCGACTT-3′;

P27 forward, 5′-GCACTGCAGAGACATGGAAG-3′;

Cyclin D1 forward, 5′-CCCTCGGTGTCCTACTTCAA-3′;

and U6 forward: 5′-CTCGCTTCGGCAGCACA-3′.

### Cell proliferation assay

CRC cells (2 × 10^3^) were seeded into 96-well plates. Cell counting kit-8 (CCK-8; DOJINDO, Japan) assay was performed following the manufacturer’s instructions. Absorbance at 450 nm was measured 1 h after the addition of 10 μl of CCK-8 reagent per well to calculate the number of viable cells every 24 h over a 96 h period. All experiments were performed in triplicate. CRC cells were plated on a 6-well plate (500 cells per well) and cultured for 10 days. The colonies were stained with 1.0% crystal violet for 5 min after fixation with 10% formaldehyde for 15 min.

### CFSE staining

CRC cells were washed and resuspended with RPMI1640 at a final concentration of 1 × 10^6^ cells/ml. CRC cells were labeled with 5 Mmcarboxy-fluorescein succinimidyl ester (CFSE, Invitrogen) for 10 min at 37 °C. The labeling reaction was quenched by addition of cold RPMI-1640 with 10% FBS and incubation on ice for 10 min. CFSE-labeled cells were washed with culture medium twice and seeded into 24-well plates. Labeled cells were harvested at indicated time points and cell proliferation was determined by FACS.

### Dual-luciferase reporter assay

293T cells were seeded in a 24-well plate and co-transfected with 0.5 μg pmirGLO vector, 80 nM of miR-760-mimic and 1 μl of Lipo3000 (Invitrogen) in 50 μl of Opti-MEM Reduced-Serum Medium (Invitrogen). NC mimic was used as the control. To verify the activation of the cyclin D1 promoter, TRE-containing sequence (5′-AAAATGAGTCAGAA-3′) was cloned into pGL4.27 vector to produce the plasmid pGL4.27-Cyclin D1-wild-type (WT). miR-760 overexpressed in SW620 or HCT116 cells, and control psico-transducted cells were seeded separately in 24-well plates and co-transfected with 0.25 μg pGL4.27-cyclinD1-WT plasmids, 0.25 μg pRL-TK plasmids (Promega), and 1 μl Lipo3000 (Invitrogen) in 50 μl Opti-MEM Reduced-Serum Medium following the manufacturer’s instructions. Twenty-four hours following transfection, the activities of Firefly and Renilla luciferases in cell lysates were measured using the Dual-Glo®Luciferase Assay System (Promega) and the Fluoroskan Ascent FL (Thermo Fischer Scientific). Firefly luciferase activity was normalized to Renilla luciferase activity. All transfection experiments were conducted in triplicate.

### Tumor formation in BALB/c nude mice

BALB/c athymic nude mice (female, 4–6 weeks old and 16–20 g) were purchased from Beijing Vital River Laboratory (Beijing, China). All animal experiments were carried out in accordance with the Guide for the Care and Use of Laboratory Animals of Zhengzhou University. To establish the CRC cancer xenograft model, the mice were randomly divided into two groups (*n* = 6 each). SW620-psico or SW620-miR-760 cells (5 × 10^6^) were suspended in 150 μl PBS and inoculated subcutaneously into the flanks of the nude mice. Tumor dimension was measured by length (L) and width (W) with a caliper twice a week, and the volumes were calculated using the formula (L × W^2^)/2. Mice were sacrificed by cervical dislocation after being anaesthetized with 10% chloral hydrate at day 31, and the tumors were excised and snap-frozen for protein and RNA extraction. IHC of the tumor tissues was performed as described below.

### IHC

Formalin-fixed, paraffin-embedded sections (3 mm) were deparaffinized in xylene, rehydrated with an alcohol gradient, and washed briefly in tap water. Endogenous peroxidase was blocked with methanol containing 0.3% hydrogen peroxide for 30 min. To retrieve antigenicity, sections were boiled in 10 mM citrate buffer (pH 5.8) for 30 min in a microwave (800 W). Next, sections were incubated with goat serum diluted in PBS (pH 7.4) for 30 min at 22 °C. Subsequently, sections were incubated at 4 °C overnight with primary antibodies specific for BATF3 diluted at 1:200 (Abcam, Cambridge, UK). The following day, sections were rinsed with fresh PBS and incubated with horseradish peroxidase-linked secondary antibodies at room temperature for 30 min. Finally, sections were stained with 3,30-diaminobenzidine (DAB) substrate (Dako, Carpinteria, CA, USA) and counterstained with Mayer’s hematoxylin. Images were recorded using a microscope (Leica, Wetzlar, Germany).

### Statistical analysis

Data analysis was performed using SPSS 19.0 statistical software or GraphPad Prism 7 software. Data were expressed as the mean ± standard deviation(SD) of at least 3 replicated experiments. Student’s t-test was performed to analyze the differences between two groups with normally distributed continuous variables. Otherwise, the Mann-Whitney U test would be used. Pearson’s coefficient correlation or linear regression analysis was used to analyze the relationships between the expression levels of specific genes. The Kaplan-Meier method was used to establish survival curves, and the survival differences were compared using the log-rank test. In all cases, a two-tailed *P* value< 0.05 was considered statistically significant.

## Results

### Low miR-760 expression was observed in CRC tissues and was associated with poor prognosis

**Fig. 1:**
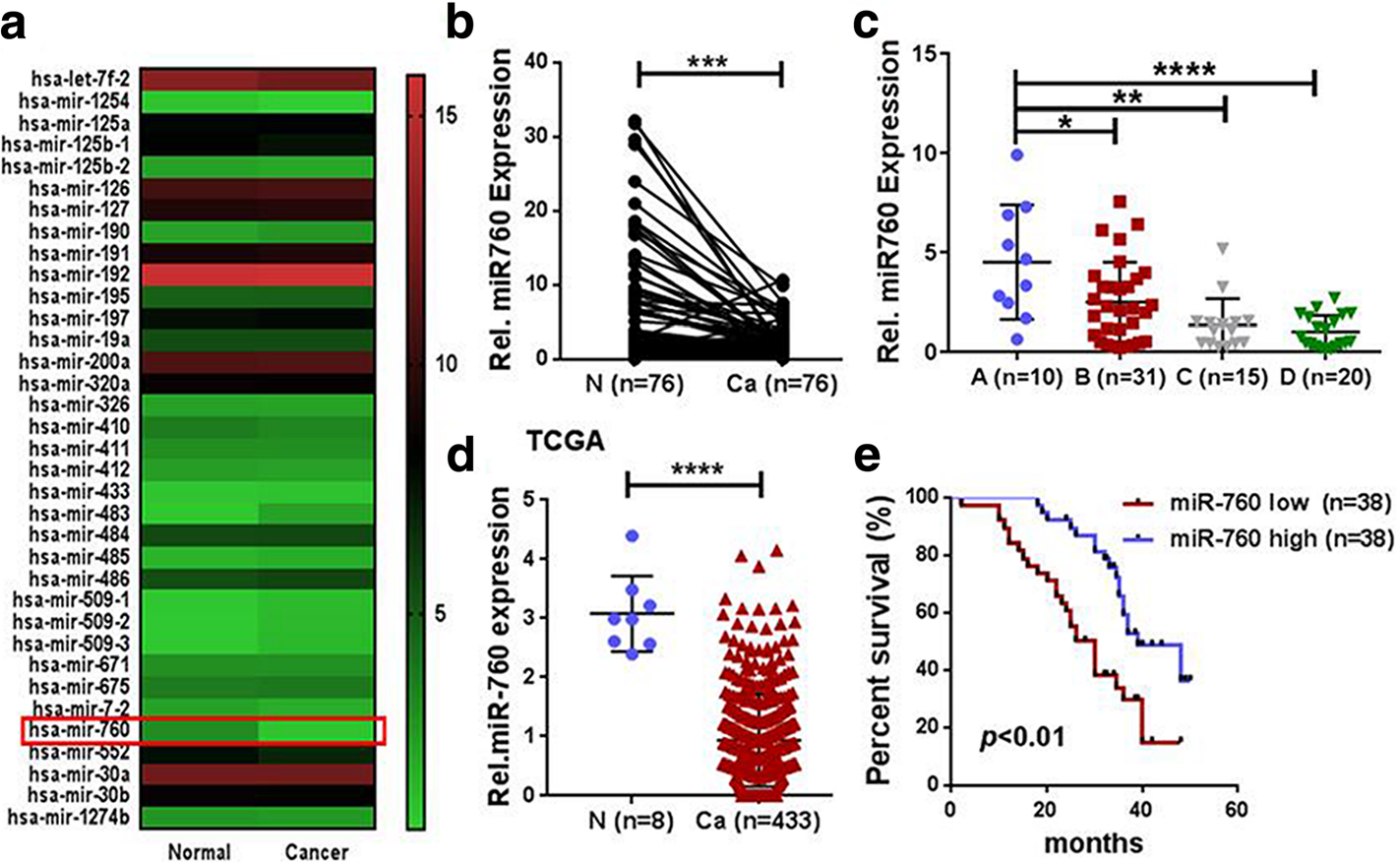
Expression of miR-760 was decreased in colorectal cancer tissues and cell lines. **a** Thirty-five miRNAs were differentially expressed between normal and cancer samples with available miRNA data in the TCGA database. **b** Real-time PCR analysis of miR-760 expression in paired fresh tissues from 76 CRC patients. Average miR-760 expression was normalized using U6 expression. ***P < 0.001. **c** Samples were divided according to their Dukes stage and the expression of miR-760 in CRC tissues was analyzed. **d** Expression profiling of miRNAsreveals that miR-760 is lower in tumor tissues compared with normal tissues (*n* = 441, *P* < 0.0001; TCGA). **e** Overall survival of 76 CRC patients who was stratified based on low expression (<median, *n* = 38) and high expression (>median, *n* = 38) of miR-760. miR-760 downregulation was significantly correlated with poor overall survival (*P* < 0.01). Error bar represents the mean ± SD of three independent experiments. ***P < 0.001, ****P < 0.0001

It has been demonstrated that miRNAs are key regulators of many cellular processes such as development, differentiation, apoptosis, and proliferation, and that several miRNAs can cooperatively induce cellular senescence and apoptosis in colorectal cancer [26]. In this regard, we screened the TCGA database, which contains data from 433 CRC tissues and 8 normal colon tissues, to identify miRNAs that are downregulated in CRC tissues. We observed that a total of 35 miRNAs were differentially expressed between normal and tumor samples. Among these miRNAs, miR-760 was identified to be the most significantly downregulated gene in CRC tissues (Fig. 1a). Next, we measured mature miR-760 expression levels in a paired independent validation sample cohort (*n* = 76) by qRT-PCR and normalized the results to U6 expression. The results showed that miR-760 expression in tumors was significantly (*P* < 0.05) reduced compared to distant healthy tissues (Fig. 1b). Moreover, the expression of miR-760 decreased in a manner that correlated with Dukes stage, where the lowest expression of miR-760 was observed in later stages including both Dukes stage C and stage D patients (Fig. 1c). Our data was further validated using the TCGA database (Fig. 1d). Together, these results suggest that miR-760 expression is decreased in human CRC cells.

To investigate whether downregulated miR-760 is involved in CRC progression, we examined the correlation between miR-760 level and the clinicopathological features of 76 patients with CRC. Statistical analyses showed that lower miR-760 expression was significantly associated with more advanced Dukes stages (*P* = 0.037) and lymph node metastasis (*P* = 0.036; Table 1). Additionally, Kaplan-Meier and Cox’s proportional hazards regression model survival analysis revealed that patients with low expression levels of miR-760 had shorter overall survival (Fig. 1e). Given the results of the integrated analysis, we hypothesize that decreased expression of miR-760 may promote CRC progression and development.

**Table 1:** Association between CRC patient characteristics and miR-760 expression

Pathological variables	Number of patients in each group	MiR-760 expression		*p* value^a^
		Low	High	
All cases	76	38	38	
Ages				0.16867
< 60	38	16 (42%)	22 (58%)	
≧ 60	38	22 (58%)	16 (42%)	
Gender				0.06046
Male	46	19 (41%)	27 (59%)	
Female	30	19 (63%)	11 (37%)	
Dukes stage				***0.03726***
A + B	43	17 (40%)	26 (60%)	
C + D	33	21 (64%)	12 (36%)	
Liver Metastasis				0.17467
M0	66	35 (53%)	31 (47%)	
M1	10	3 (30%)	7 (70%)	
Cancer Type				0.15364
Colon	28	11 (39%)	17 (61%)	
Recta	48	27 (56%)	21 (44%)	
Lymph Node Metastasis				***0.03567***
N0	45	18 (40%)	27 (60%)	
N1	31	20 (65%)	11 (35%)	
Preoperative serum CEA level				0.24319
< 5 μg/mL	45	20 (44%)	25 (56%)	
≥ 5 μg/mL	31	18 (58%)	13 (42%)	

^a^The data were subjected to Cox’s proportional hazards regression model. Bold italics indicate statistically significant values (*P* < 0.05)

### MiR-760 inhibited proliferation and colony formation of CRC cells

**Fig. 2:**
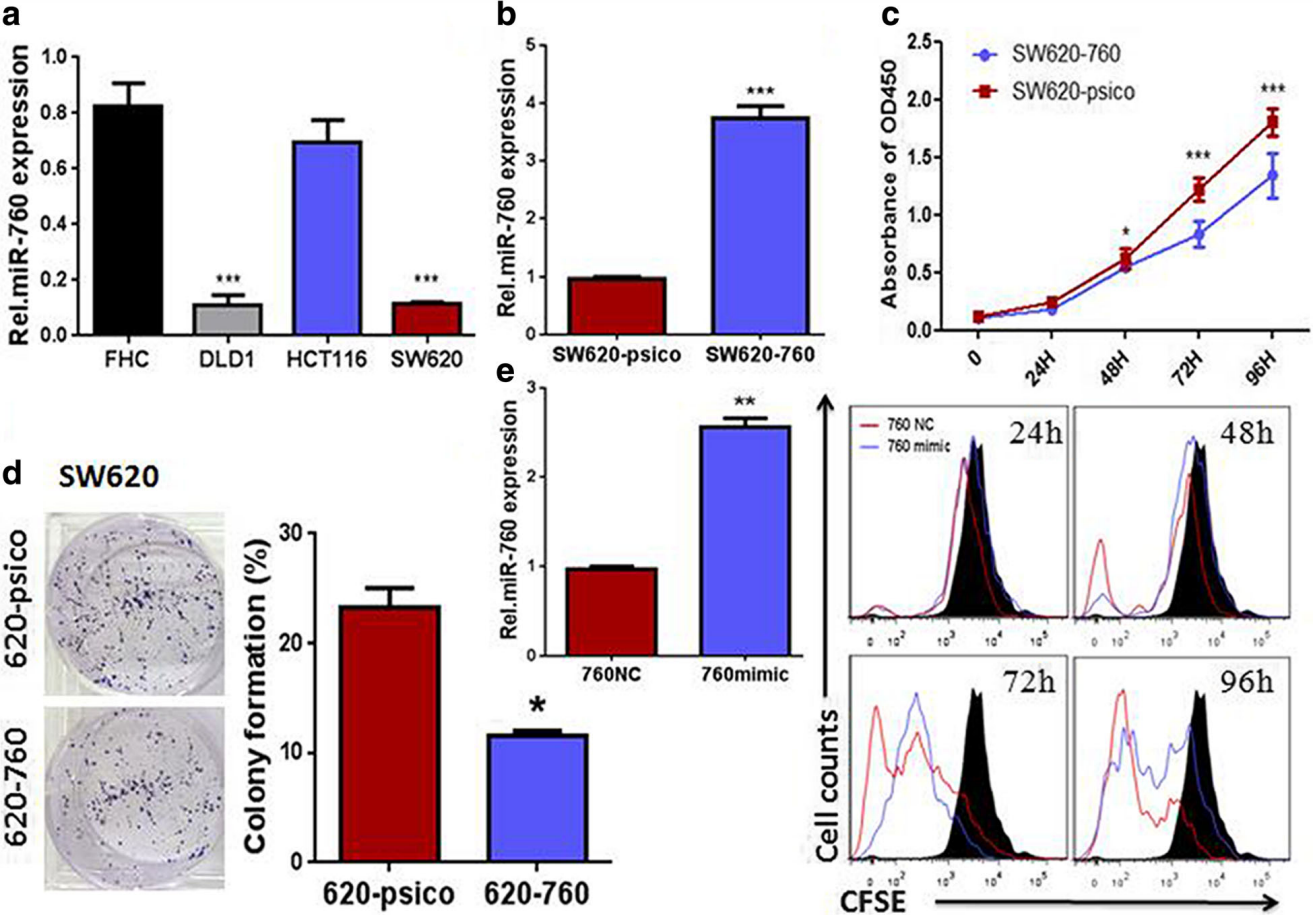
Upregulation of miR-760 inhibited colorectal cancer cell proliferation. **a** miR-760 expression in human colorectal mucosa cell line FHC, and in CRC cell lines, including DLD1, HCT116, and SW620. **b** Real-time PCR analysis of miR-760 expression in SW620 cells expressing miR-760 and control cells. **c** Effects of miR-760 on the proliferation of SW620 cells lines analyzed by the CCK-8 assay. **d** Representative micrographs (left) and quantification (right) of crystal violet-stained cell colonies formed by the indicated CRC cell lines 10 days after inoculation. **e** (left) Real-time PCR analysis of miR-760 expression in SW620 cells transfected with miR-760 mimic or negative control. (right) CFSE staining reveals proliferation of SW620 cells transfected with miR-760 or vector. Error bar represents the mean ± SD of three independent experiments. *P < 0.05, **P < 0.01, ***P < 0.001

As miR-760 had a consistent pattern of downregulation in human CRC samples, we examined the expression pattern of miR-760 in CRC cell lines. We found lower expression of miR-760 in SW620 and DLD1 cell lines compared to miR-760 expression in HCT116 cells and the normal intestinal epithelium cell line FHC (Fig. 2a). To investigate the effect of miR-760 on the development and progression of CRC, gain-of-function studies using psico-miR-760 were performed to investigate whether miR-760 helped to suppress CRC cell proliferation (Fig. 2b). CCK-8 and colony formation assays revealed that overexpression of miR-760 dramatically decreased the growth rate of CRC cells (Fig. 2c and d). Furthermore, a CFSE staining assay was used to verify the role of miR-760 on the growth of CRC cell line SW620. Because CFSE and GFP are detected through the same fluorescent channel, we used miR-760-mimic transduction to overexpress miR-760 (Fig. 2e, left). We observed that growth rate was substantially decreased in miR-760-overexpressing CRC cells compared with the growth rate of control cells (Fig. 2e, right). These results suggest that upregulation of miR-760 suppresses the proliferation of CRC cells.

**Fig. 3:**
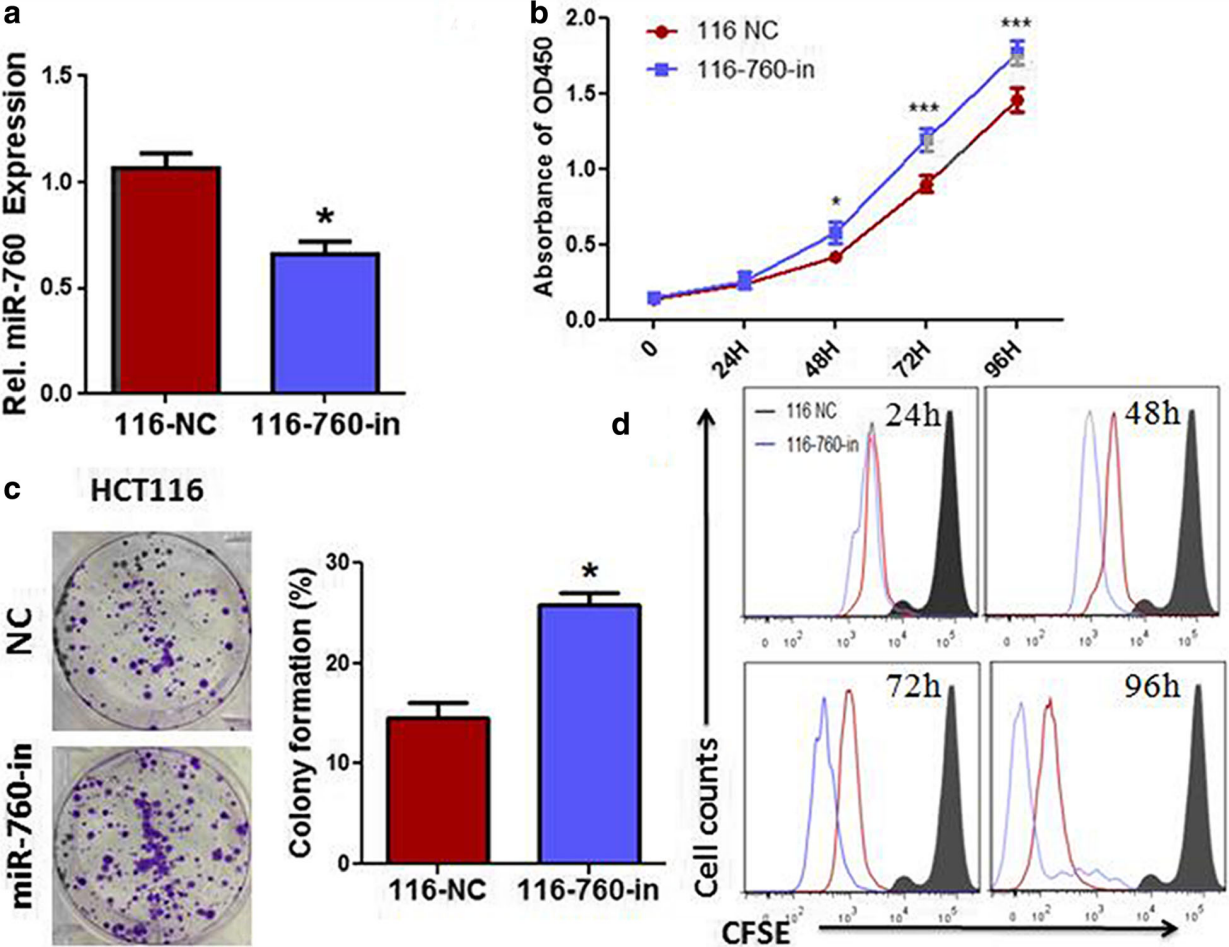
Inhibition of miR-760 promoted the proliferation of colorectal cancer cells. **a** Real-time PCR analysis of miR-760 expression in HCT116 cells transfected with miR-760 inhibitor (116-760-in) or miRNA inhibitor negative control (116-NC). **b** The proliferation of CRC cell line HCT116 transfected with miR-760 inhibitor and negative control measured by the CCK-8 assay. **c** Representative micrographs (left) and quantification (right) of crystal violet-stained cell colonies formed by the indicated CRC cell lines 10 days after inoculation. **d** CFSE staining reveals proliferation of HCT116 cells transfected with miR-760 inhibitor or negative control. Error bar represents the mean ± SD of three independent experiments. *P < 0.05, ***P < 0.001

We performed Lose-of-function studies using a miR-760 inhibitor to further investigate if miR-760 suppressed CRC cell proliferation (Fig. 3a). As shown in Fig. 3b and c, suppression of miR-760 by transfection with the miR-760 inhibitor significantly increased the growth rate of CRC cell lines when compared with cells innegative control (NC) group. Additionally, CFSE staining showed the proliferation of miR-760-inhibitor-transfected HCT116 cells increased compared to that of NC-transfected cells (Fig. 3d). These results suggest that downregulation of miR-760 promotes the proliferation of CRC cells.

### MiR-760 targeted human BATF3 and inhibited cyclinD1 in CRC cells

**Fig. 4:**
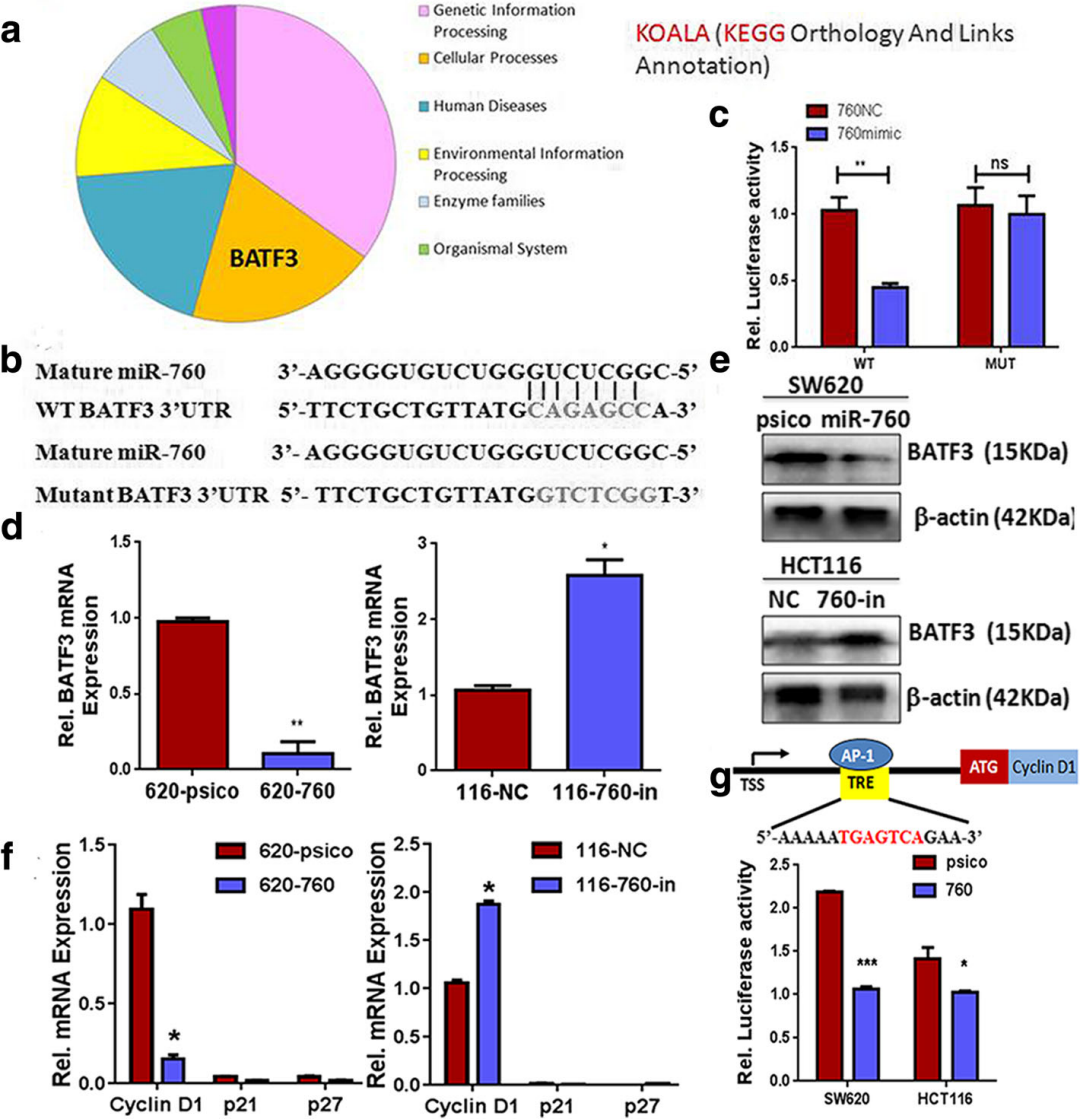
miR-760 directly targeted the 3′-UTR of BATF3 mRNA. **a** Annotation of predicted top 50 target genes using KOALA (http://www.kegg.jp/blastkoala/). BATF3 was revealed as a potential target related to cellular processes. **b** Schematic representation of miR-760 target sites in the 3′-UTR of BATF3 mRNA and a miR-760 mutant containing seven altered nucleotides in the seed sequence (mutant BATF33′-UTR). **c** Luciferase assay of pmir-GLO reporter plasmids carrying wild-type or mutant BATF3 3′-UTR co-transfected with miR-760 mimic (50 nM, 760 mimic) or negative control (50 nM, 760NC) in 293 T cells. Expression of BATF3 mRNA (**d**) and protein (**e**) in SW620 (**d** left and **e** upper) and HCT116 cells (**d** right and **e** bottom) transfected with the indicated miRNAs. **f** Real-time PCR analysis of cyclinD1, p21, and p27 mRNA expression in SW620 cells (left) and HCT116 (right). **g** (upper) The AP-1 binding site is located at 1.1 kb downstream of the cyclin D1 transcription start site (TSS). (bottom) miR-760-overexpressed SW620 or HCT116 cells were transfected with the wild-type (WT) cyclin D1 promoter-luciferase reporter plasmids and expression was assessed by the luciferase assay. Error bar represents the mean ± SD of three independent experiments. *P < 0.05, **P < 0.01, ***P < 0.001

Upon finding that miR-760 inhibits the proliferation of CRC cells, we next explored the possible mechanisms behind this function. We used the publicly available algorithm TargetScan (v7.1)—a public database of microRNAs and their targets—to predict the target(s) of miR-760 in humans. The top 50 predicted target genes were analyzed using a freely available Web site [27] (an automatic annotation server for genome and metagenome sequences http://www.kegg.jp/blastkoala/) to screen for genes related to cellular processes. This analysis revealed BATF3 as a potential target of miR-760 (Fig. 4a). To test if BATF3 expression is regulated by miR-760, we cloned the BATF3 3′-UTR into a luciferase reporter plasmid pmir-GLO and quantified expression of the adjacent hRluc coding region. BATF3 harbors conserved miR-760 cognate binding sites, namely WT BATF33′-UTR (Fig. 4b). The results showed that miR-760 suppressed luciferase activity for the reporter plasmid carrying WT BATF3 3′-UTR, but no significant effects were observed for the reporter plasmid carrying mutant BATF3 3′-UTR (Fig. 4c, *P*< 0.05). These results suggest that miR-760 binds directly to the predicted binding site(s) in the BATF3 3′-UTR and negatively regulates BATF3 expression. To confirm the direct regulation of miR-760 on BATF3 expression, we detected BATF3 expression in SW620-760 and HCT116 cells transfected with miR-760 inhibitor. The results from both qRT-PCR (Fig. 4d) and western blot (Fig. 4e) revealed that the BATF3 expression level was reduced in miR-760-overexpressing cells, and its level was restored upon treatment with miR-760 inhibitor. Interestingly, the expression of another AP-1 member c-Jun had similar tendency to BATF3 (Additional file 2: Figure S4a and b). As c-Jun was not in the predictive list of miR-760, we concluded that miR-760 directly targets BATF3 in CRC cells.

AP-1 transcription factors have been shown to play numerous roles in cell growth and proliferation. In particular, BATF3 can dimerize with Jun in the MYC promoter and support the proliferation and survival of lymphoma cells [28]. Therefore, we further examined the expression of three proliferation-associated genes, p21, p27, and cyclin D1. However, p21 and p27 mRNAs were too low to be detected in SW620, HCT116, and other CRC cells (Fig. 4f and Additional file 2: Figure S4c). Furthermore, the results showed that cyclin D1 mRNA was significantly downregulated by ectopic miR-760, whereas it was upregulated by inhibition of miR-760 (Fig. 4f). To determine whether BATF3 or AP-1 regulates cyclin D1 at the transcriptional level, we used a dual luciferase reporter gene promoter assay in which the cyclin D1 promoter was cloned upstream to the luciferase gene. The potential TRE-like sequence (5′-AAAATGAGTCAGAA-3′) is located at 1.1 kb downstream from the transcription start site (TSS; Fig. 4g). Ectopic miR-760 resulted in a reduction of luciferase activity by approximately 50% in SW620 cells and 30% in HCT116 cells compared with cells in the psico-transducted group (Fig. 4g). Collectively, these results reveal that miR-760 directly targets BATF3 and inhibits cyclinD1 via AP-1 in CRC cells.

### BATF3 suppression was critical for miR-760-induced cell proliferation in CRC cells

**Fig. 5:**
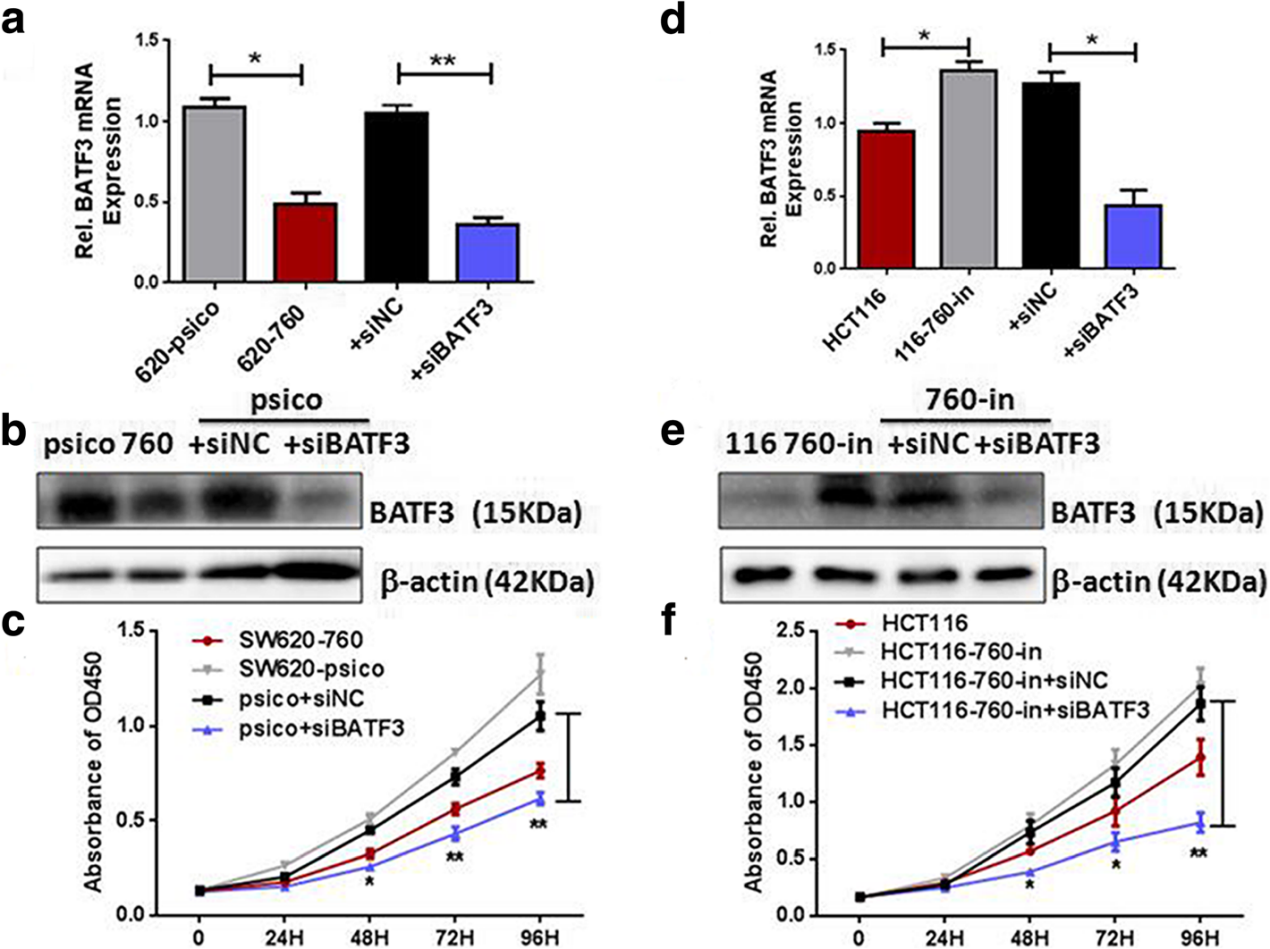
miR-760 inhibited CRC cell progression by inhibiting BATF3. The expression levels of BATF3 in SW620-psico (**a**, **b**), and HCT116-miR-760-inhibitor cells (**d**, **e**) which were transfected with BATF3-siRNA, as measured by Real-time PCR (**a**, **d**) and western blotting (**b**, **e**). β-actin served as the loading control. CCK-8 assay indicated proliferation of SW620-psico (**c**), and HCT116-miR-760-inhibitor cells (**f**) transfected with BATF3-siRNA were significantly inhibited compared with negative control (siNC). Error bars represent mean ± SD from three independent experiments. *P < 0.05, **P < 0.01

To further investigate the effect of BATF3 reduction on CRC progression, we suppressed endogenous BATF3 expression using a BATF3-specific siRNA in both SW620-psico (Fig. 5a,b) and miR-760 inhibitor-transfected HCT116 cells (Fig. 5d,e). Results from the CCK-8 assay revealed that silencing BATF3 in SW620-psico and miR-760 inhibitor-transfected HCT116 cells increased the growth rate of the cells (Fig. 5c, f). The results suggest that silencing BATF3 expression in SW620-psico and miR-760 inhibitor-transfected HCT116 cells can reverse the effect of the miR-760 inhibitor on HCT116 cell proliferation. These results confirmed that miR-760 inhibits CRC cell proliferation by suppressing endogenous BATF3 expression and BATF3 plays an important role in miR-760-mediated repression of CRC cell proliferation.

### MiR-760 suppressed tumor growth in vivo

**Fig. 6:**
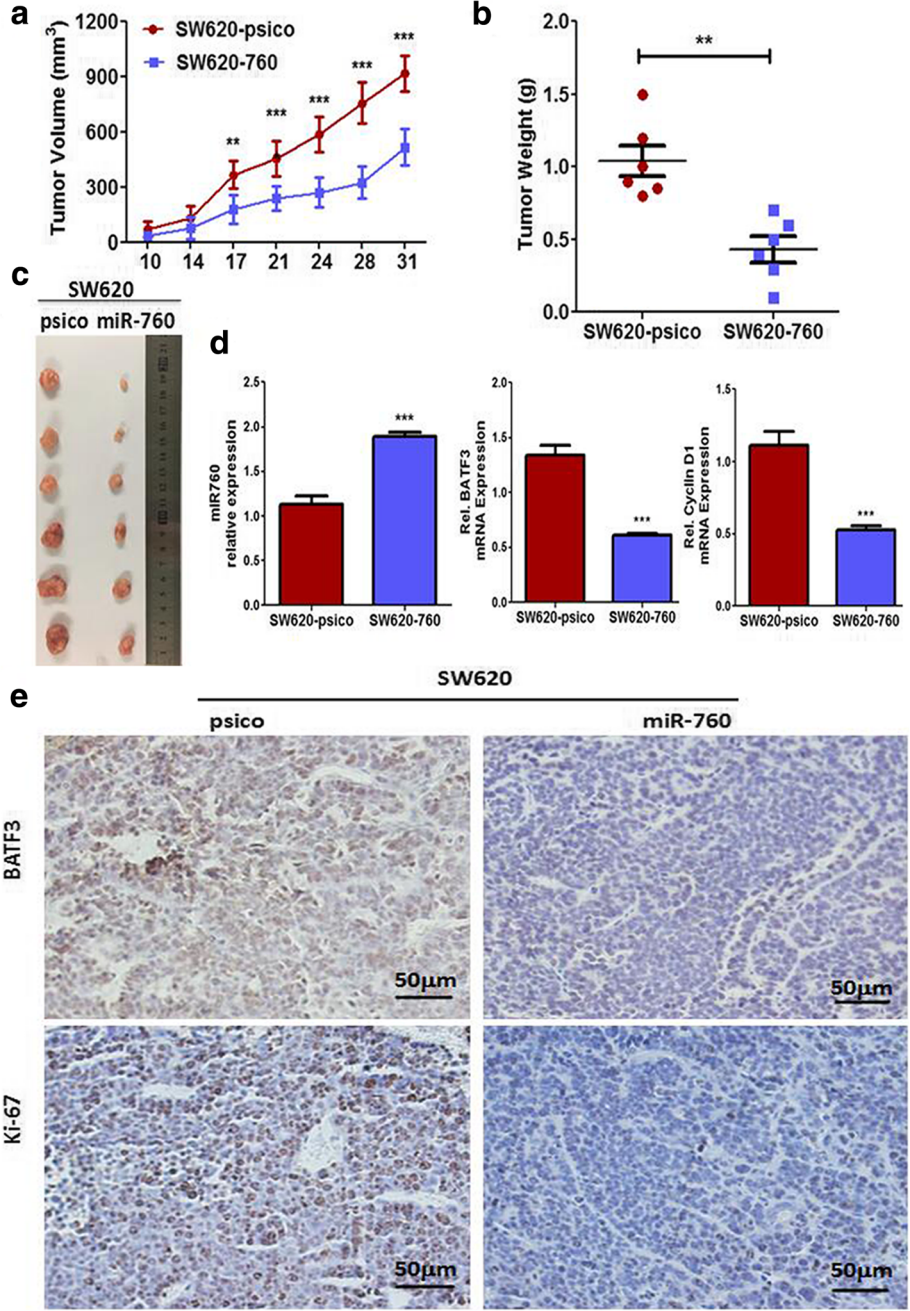
Ectopic expression of miR-760 suppressed tumor growth in vivo. The volumes (**a**) and weights (**b**) of the tumors grown in the xenograft mouse model. Data are presented as mean ± SD (*n* = 6). **c** Photographs of the dissected tumors from miR-760-overexpressed group and control group. **d** Expression of miR-760, BATF3 and cyclin D1 mRNA in tumors. **e** Immunohistochemistry of tumor tissues in SW620-psico and SW620-760 group. Assays were performed in triplicates. Mean ± SD is shown. Statistical analysis was conducted using Student’s t test. **P < 0.01, ***P < 0.001

To confirm the tumor suppressive role of miR-760 in vivo, we established a BALB/c nude mouse xenograft model using SW620 cells. We evaluated in vivo tumor growth by measuring tumor volume and weight. Mice with SW620-miR-760 tumors had smaller tumor size (*P* < 0.001, Fig. 6a) and less tumor weight (*P* < 0.01, Fig. 6b) than those with SW620-psico tumors (Fig. 6a, c). Therefore, miR-760 significantly inhibits the tumorigenesis of SW620 cells in vivo. We further examined the expression of BATF3 and Ki67 in the subcutaneous tumor samples. Consistent with the proposed regulation of BATF3 and CRC cell proliferation by miR-760, expression patterns of BATF3 and Ki67 in SW620-miR-760 tumors were similar to the in vitro results, supporting the tumor suppressor function of miR-760 in CRC tumorigenesis (Fig. 6d, 6d). These results indicate that miR-760 inhibits tumor progression in vivo by targeting BATF3 and downstream cyclin D1-induced proliferation.

### Clinical relevance of miR-760 and BATF3 expression in CRC

**Fig. 7:**
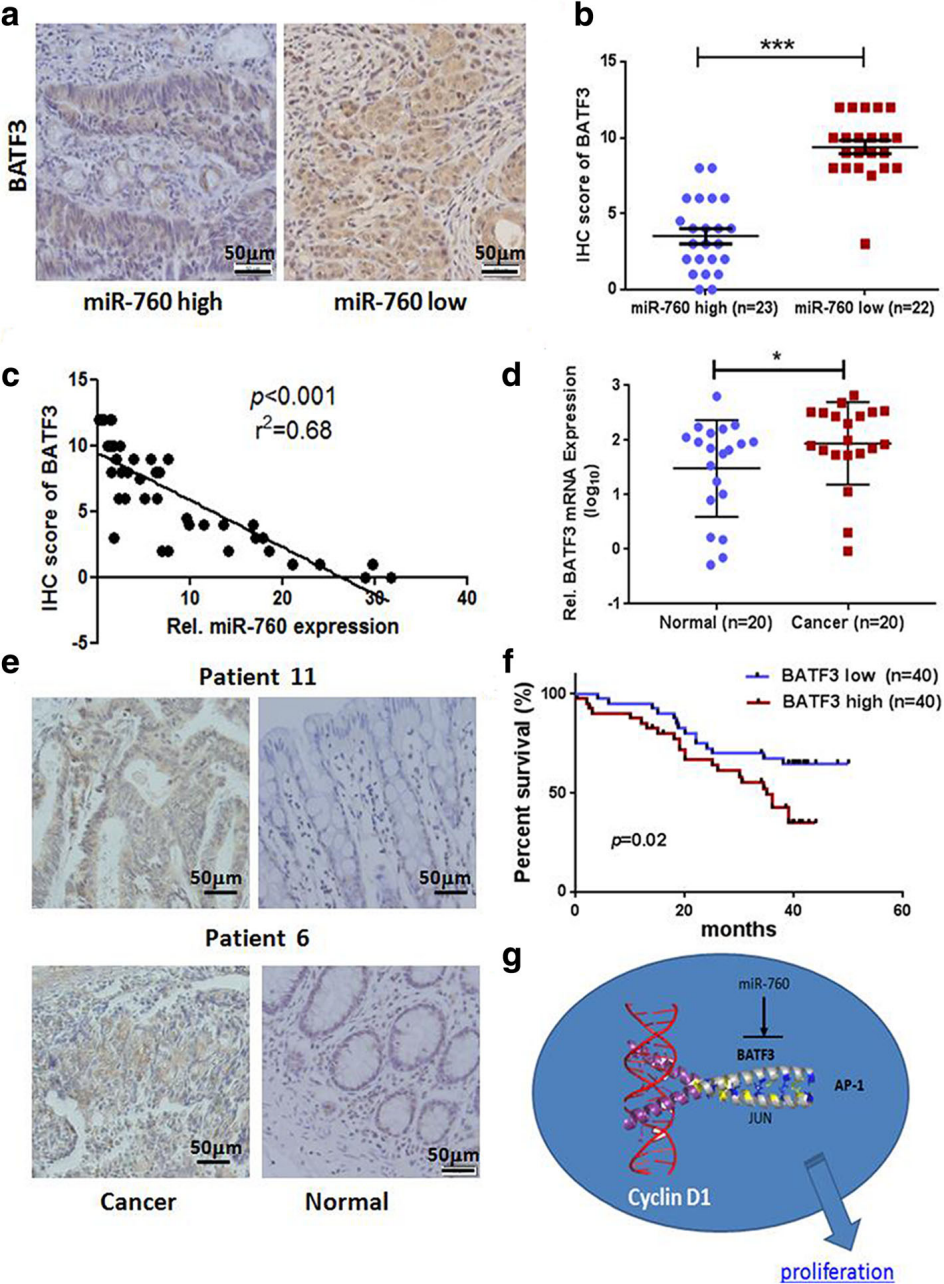
The relationships between miR-760 expression and the expression of BATF3 in human CRC tissue. **a** BATF3 expression in fixed CRC tissues was examined using immunohistochemistry. Representative images are shown (× 200). **b** Patients with high miR-760 expression had significantly lower BATF3 expression compared with those with low miR-760 expression (*P* < 0.001). **c** miR-760 was inversely associated with intratumoral BATF3 expression (*r*^2^ = 0.68, *P* < 0.001). **d** Real-time PCR analysis of BATF3 expression in 20 paired CRC tissues. **e** Representative images of BATF3 staining are shown in CRC tissues and matched normal tissues (× 200). **f** Overall survival of patients with CRC cancer was stratified according to low expression (<median, *n* = 40) and high expression (>median, *n* = 40) of BATF3. BATF3 upregulation was significantly correlated with poor overall survival (*P* = 0.02). **g** Schematic diagram of the proposed molecular mechanismby which miR-760 modulates CRC cell proliferation

To investigate the relationships between miR-760 and BATF3, we examined BATF3 expression in CRC tissues using IHC and representative images are shown in Fig. 7a. Using a median value of miR-760 expression as a cutoff point, we divided the samples obtained from 45 CRC patients with available miR-760 data into two groups. Patients with higher miR-760 expression had significantly lower BATF3 expression compared to those with lower miR-760 expression (*P* < 0.001, Fig. 7b). In addition, BATF3 expression was found to be inversely correlated with miR-760 expression (*r*^2^ = 0.68,*P* < 0.001, Fig. 7c). The results further support an inverse relationship between miR-760 and BATF3 expression in human CRC tissue.

Moreover, we analyzed BATF3 expression levels in paired CRC samples randomly selected from previous 45 patients (*n* = 20) using qRT-PCR and IHC. The results showed that BATF3 expression in tumor tissues was significantly (*P* < 0.05) higher than distant normal tissues (Fig. 7d). Accordingly, we found that BATF3 is mainly expressed in cancerous cells, whereas there was no positive staining observed in normal colorectal epithelial cells (Fig. 7e). Kaplan-Meier and Cox’s proportional hazards regression model survival analysis revealed that patients with high expression levels of BATF3 (median IHC score of BATF3 as cutoff point) had poor overall survival (*P* = 0.02, Fig. 7f). Taken together, BATF3 is upregulated in human CRC and miR-760 is reversely related with BATF3 expression in CRC tissues.

## Discussion

In the present study, we found that miR-760 is dramatically downregulated in human CRC tissues compared with normal colorectal tissues. Moreover, upregulation of miR-760 suppresses proliferation of colorectal cancer cells by targeting BATF3 3′-UTR. The negative regulation of BATF3 by miR-760 leads to downregulation of cyclinD1 in CRC cells. Collectively, miR-760 suppresses tumor cell growth in vitro and tumorigenesis in vivo. Our study may serve as a rational for targeting the miR-760/BATF3 interaction in a novel therapeutic application to treat CRC patients.

miRNAs are a class of non-coding RNA molecules that play a vital role in cell differentiation, proliferation, and survival by binding to complementary target mRNAs, resulting in mRNA translational inhibition or degradation [29]. Thus, miRNA mimics and miRNA inhibitors (antimiRs) have shown promise in preclinical development [30]. It has been reported that miR-760 is downregulated in CRC plasma, and has an 80.0% sensitivity and 72.4% specificity (AUC = 0.788) for the early detection of CRC, suggesting it can be used for early CRC diagnosis [31]. In the present study, we found that miR-760 expression in tumor tissues was significantly reduced compared with healthy cells, and low miR-760 expression was associated with advanced Dukes stage and lymph node metastasis (Table 1). Moreover, low miR-760 expression can predict poorer outcomes in CRC patients. Taken together, the expression of miR-760 in tumors might serve as a prognostic marker for CRC patients.

miRNAs are known to play an important role in human tumorigenesis by altering the expression of multiple genes, therefore, elucidating the molecular mechanism by which miRNAs function in tumor development may provide valuable diagnostic and therapeutic strategies for malignancy [32–34]. It has been shown that miR-760 was downregulated in chemoresistant breast cancer tissues and might mediate breast cancer chemoresistance via the regulation of p-gp expression [35]. Moreover, miR-760 was found to reduce the cancer stem cell population and inhibit breast cancer cell proliferation and metastasis via inactivation of NANOG transcription factor [36]. However, there is also evidence to suggest that miR-760 acts as an onco-miR in certain cancer types. For example, it is reported that miR-760 was strongly overexpressed in ovarian cancer and miR-760 upregulation drastically promoted ovarian cancer cell proliferation by inhibiting the expression of PHLPP2 [37]. Herein, we found that miR-760 was downregulated in CRC tissues and that miR-760 overexpression suppressed CRC cell proliferation by repressing BATF3 expression. As there are few reports that have explored the function of miR-760 in cancer and the precise role of miR-760 in cancer pathogenesis and progression remains unclear and even controversial, more studies are needed to determine the exact role and mechanism of miR-760 in tumor development.

In normal cells, BATF3 (also known as JDP1 and p21SNFT) is expressed in T helper type 1 cells and plays a major role in the development and function of conventional dendritic cells [20]. Thanks to its leucine zipper motif sequence, BATF3 was recognized as belonging to a class of proteins that heterodimerize with JUN proteins [38, 39]. The leucine zipper motif of BATF3 has been shown to be involved in the dimerization of the bZIP proteins to generate composite transcription factors that recognize palindromic TPA response elements (TREs) in their target genes [40, 41]. Although BATF3 molecules lack transcriptional activation domains, unlike the AP-1 factors FOS and JUN, it has been reported that BATF3 can exert unique positive transcriptional specificity through interacting with members of the interferon regulatory factor (IRF) family [24]. Recently, it has been reported that BATF3 forms AP-1 complexes with JUN in the MYC promoter and controls MYC expression in cHL (classical Hodgkin lymphoma) and ALCL (anaplastic large cell lymphoma) cell lines, critically supporting their proliferation and survival [28]. However, there are a few conflicting reports showing that BATF3 might play a positive regulatory role in other tumor cells. Here, we observed miR-760 directly targeted BATF3 and ectopic miR-760 resulted in a reduction of BATF3 and cyclinD1.

It has been previously shown that the JNK-mediated phosphorylation of AP-1 (c-Jun) upregulates cyclin D1 to drive proliferation in liver cell regeneration and mouse epidermal cell transformation [42, 43], which supported our data that miR-760 inhibited cyclinD1 via AP-1 in CRC cells. Experimentally, we verified that cyclin D1 mRNA 3′-UTR had AP-1(c-Jun)-binding sites in both SW620 and HCT116 CRC cells. Interestingly, we also found that loss of miR-760 accelerated the expression of BATF3, c-Jun (Additional file 2: Figure S4), cyclin D1 (Fig. 4f) and vice versa. Therefore, we propose that BATF3 forms AP-1 complexes with c-Jun in the cyclin D1 promoter and controls cyclin D1 expression in CRC cell lines, consequently supporting CRC cell proliferation and survival. As for the mechanism how BATF3 dimers with c-Jun and the complicated network between BATF3 and other AP1 family members like c-Jun, we will further explore the complex members of AP1 and their function or clinical significance in CRC in the future. Therefore, cyclin D1 may be tightly regulated by miR-760 in CRC, and miR-760 restoration could target BATF3/AP-1/cyclin D1 pathway to suppress CRC progression. Furthermore, it is likely that other molecules or signaling pathways will be discovered that are also targeted by miR-760 in CRC. Future work will focus on revealing additional functions of miR-760 in CRC carcinogenesis and progression.

## Conclusion

In summary, for the first time, our mechanistic study revealed BATF3 and Cyclin D1 genes as targets for miR-760, which underlies its role in CRC development. miR-760 substantially suppressed CRC cell proliferation by inhibiting the expression of BATF3 and cyclin D1. In view of the anti-proliferation effects ofmiR-760 on tumor cells, it would be worth investigating the use of miR-760 as a therapeutic agent to treat CRC.

## Additional files

Additional file 1: **Table S1.** The groups of CRC patient population used in different figures. (DOC 29 kb)

Additional file 2: **Figure S4.** MiR-760 inhibited human BATF3/c-Jun and downstream cyclin D1 in CRC cells. (DOCX 109 kb)

## Abbreviations

3’-UTR: 3′-untranslated region
AP-1: Activator protein 1
BATF3: Basic leucine zipper transcriptional factor ATF-like 3
CRC: Colorectal cancer
FCM: Flow cytometry method
IHC: Immunohistochemistry
NC: Negative control
qRT-PCR: Quantitative reverse transcription PCR
TCGA: The Cancer Genome Atlas

## References

[1] Siegel RL, Miller KD, Fedewa SA, Ahnen DJ, Meester RGS, Barzi A, Jemal A (2017). Colorectal cancer statistics, 2017. CA Cancer J Clin.

[2] Chakradhar S (2015). Colorectal cancer: 5 big questions. Nature.

[3] Lu J, Getz G, Miska EA, Alvarez-Saavedra E, Lamb J, Peck D (2005). MicroRNA expression profiles classify human cancers. Nature.

[4] Baek D, Villen J, Shin C, Camargo FD, Gygi SP, Bartel DP (2008). The impact of microRNAs on protein output. Nature.

[5] Zhong S, Li W, Chen Z, Xu J, Zhao J (2013). MiR-222 and miR-29a contribute to the drug-resistance of breast cancer cells. Gene.

[6] Huang T, Yin L, Wu J, Gu JJ, Wu JZ, Chen D (2016). MicroRNA-19b-3p regulates nasopharyngeal carcinoma radiosensitivity by targeting TNFAIP3/NF-kappaB axis. J Exp Clin Cancer Res.

[7] Zhuang Q, Zhou T, He C, Zhang S, Qiu Y, Luo B (2016). Protein phosphatase 2A-B55delta enhances chemotherapy sensitivity of human hepatocellular carcinoma under the regulation of microRNA-133b. J Exp Clin Cancer Res.

[8] Moridikia A, Mirzaei H, Sahebkar A, Salimian J. MicroRNAs: potential candidates for diagnosis and treatment of colorectal cancer. J Cell Physiol.2018;233(2):901–13. 10.1002/jcp.25801 28092102

[9] Vogt PK (2002). Fortuitous convergences: the beginnings of JUN. Nat Rev Cancer.

[10] Sankpal NV, Mayfield JD, Willman MW, Fleming TP, Gillanders WE (2011). Activator protein 1 (AP-1) contributes to EpCAM-dependent breast cancer invasion. Breast Cancer Res.

[11] Shi M, Liu D, Duan H, Han C, Wei B, Qian L (2010). Catecholamine up-regulates MMP-7 expression by activating AP-1 and STAT3 in gastric cancer. Mol Cancer.

[12] Mahata S, Bharti AC, Shukla S, Tyagi A, Husain SA, Das BC (2011). Berberine modulates AP-1 activity to suppress HPV transcription and downstream signaling to induce growth arrest and apoptosis in cervical cancer cells. Mol Cancer.

[13] Hong H, Jiang L, Lin Y, He C, Zhu G, Du Q, Wang X, She F, Chen Y (2016). TNF-alpha promotes lymphangiogenesis and lymphatic metastasis of gallbladder cancer through the ERK1/2/AP-1/VEGF-D pathway. BMC Cancer.

[14] Eferl R, Wagner EF (2003). AP-1: a double-edged sword in tumorigenesis. Nat Rev Cancer.

[15] Zhang Y, Xu X, Zhang M, Wang X, Bai X, Li H (2016). MicroRNA-663a is downregulated in non-small cell lung cancer and inhibits proliferation and invasion by targeting JunD. BMC Cancer.

[16] Xu Z, Bu Y, Chitnis N, Koumenis C, Fuchs SY, Diehl JA (2016). miR-216b regulation of c-Jun mediates GADD153/CHOP-dependent apoptosis. Nat Commun.

[17] Aronheim A, Zandi E, Hennemann H, Elledge SJ, Karin M (1997). Isolation of an AP-1 repressor by a novel method for detecting protein-protein interactions. Mol Cell Biol.

[18] Iacobelli M, Wachsman W, McGuire KL (2000). Repression of IL-2 promoter activity by the novel basic leucine zipper p21SNFT protein. J Immunol.

[19] Echlin DR, Tae HJ, Mitin N, Taparowsky EJ (2000). B-ATF functions as a negative regulator of AP-1 mediated transcription and blocks cellular transformation by Ras and Fos. Oncogene.

[20] Hildner K, Edelson BT, Purtha WE, Diamond M, Matsushita H, Kohyama M (2008). Batf3 deficiency reveals a critical role for CD8alpha+ dendritic cells in cytotoxic T cell immunity. Science.

[21] Schraml BU, Hildner K, Ise W, Lee WL, Smith WA, Solomon B (2009). The AP-1 transcription factor Batf controls T(H)17 differentiation. Nature.

[22] Betz BC, Jordan-Williams KL, Wang C, Kang SG, Liao J, Logan MR, Kim CH, Taparowsky EJ (2010). Batf coordinates multiple aspects of B and T cell function required for normal antibody responses. J Exp Med.

[23] Ise W, Kohyama M, Schraml BU, Zhang T, Schwer B, Basu U (2011). The transcription factor BATF controls the global regulators of class-switch recombination in both B cells and T cells. Nat Immunol.

[24] Murphy TL, Tussiwand R, Murphy KM (2013). Specificity through cooperation: BATF-IRF interactions control immune-regulatory networks. Nat Rev Immunol.

[25] Liu JY, Li F, Wang LP, Chen XF, Wang D, Cao L (2015). CTL- vs Treg lymphocyte-attracting chemokines, CCL4 and CCL20, are strong reciprocal predictive markers for survival of patients with oesophageal squamous cell carcinoma. Br J Cancer.

[26] Kim SY, Lee YH, Bae YS (2012). MiR-186, miR-216b, miR-337-3p, and miR-760 cooperatively induce cellular senescence by targeting alpha subunit of protein kinase CKII in human colorectal cancer cells. Biochem Biophys Res Commun.

[27] Kanehisa M, Sato Y, Morishima K (2016). BlastKOALA and GhostKOALA: KEGG tools for functional characterization of genome and metagenome sequences. J Mol Biol.

[28] Lollies A, Hartmann S, Schneider M, Bracht T, Weiss AL, Arnolds J, et al. An oncogenic axis of STAT-mediated BATF3 upregulation causing MYC activity in classical Hodgkin lymphoma and anaplastic large cell lymphoma. Leukemia. 2017; 10.1038/leu.2017.203. 10.1038/leu.2017.203 28659618

[29] Bartel DP (2004). MicroRNAs: genomics, biogenesis, mechanism, and function. Cell.

[30] Rupaimoole R, Slack FJ (2017). MicroRNA therapeutics: towards a new era for the management of cancer and other diseases. Nat Rev Drug Discov.

[31] Yang Y, Gu X, Zhou M, Xiang J, Chen Z (2013). Serum microRNAs: a new diagnostic method for colorectal cancer. Biomed Reports.

[32] Hayes J, Peruzzi PP, Lawler S (2014). MicroRNAs in cancer: biomarkers, functions and therapy. Trends Mol Med.

[33] Elshafei A, Shaker O, Abd El-Motaal O, Salman T. The expression profiling of serum miR-92a, miR-375, and miR-760 in colorectal cancer: an Egyptian study. Tumour Biol 2017; 39:1010428317705765. 10.1177/1010428317705765 28618945

[34] Lee K, Ferguson LR (2016). MicroRNA biomarkers predicting risk, initiation and progression of colorectal cancer. World J Gastroenterol.

[35] Lv J, Xia K, Xu P, Sun E, Ma J, Gao S (2014). miRNA expression patterns in chemoresistant breast cancer tissues. Biomed Pharmacotherapy.

[36] Han ML, Wang F, Gu YT, Pei XH, Ge X, Guo GC (2016). MicroR-760 suppresses cancer stem cell subpopulation and breast cancer cell proliferation and metastasis: by down-regulating NANOG. Biomed Pharmacotherapy.

[37] Liao Y, Deng Y, Liu J, Ye Z, You Z, Yao S, He S (2016). MiR-760 overexpression promotes proliferation in ovarian cancer by downregulation of PHLPP2 expression. Gynecol Oncol.

[38] Chinenov Y, Kerppola TK (2001). Close encounters of many kinds: Fos-Jun interactions that mediate transcription regulatory specificity. Oncogene.

[39] Vinson C, Myakishev M, Acharya A, Mir AA, Moll JR, Bonovich M (2002). Classification of human B-ZIP proteins based on dimerization properties. Mol Cell Biol.

[40] Landschulz WH, Johnson PF, McKnight SL (1988). The leucine zipper: a hypothetical structure common to a new class of DNA binding proteins. Science.

[41] Vinson CR, Sigler PB, McKnight SL (1989). Scissors-grip model for DNA recognition by a family of leucine zipper proteins. Science.

[42] Schwabe RF, Bradham CA, Uehara T, Hatano E, Bennett BL, Schoonhoven R, Brenner DA (2003). c-Jun-N-terminal kinase drives cyclin D1 expression and proliferation during liver regeneration. Hepatology.

[43] Zhang D, Li J, Gao J, Huang C (2009). c-Jun/AP-1 pathway-mediated cyclin D1 expression participates in low dose arsenite-induced transformation in mouse epidermal JB6 Cl41 cells. Toxicol Appl Pharmacol.

