# Supplementary material for: MiR-760 suppresses human colorectal cancer growth by targeting BATF3/AP-1/cyclinD1 signaling
Source: J Exp Clin Cancer Res. 2018 Apr 16;37:83. doi: 10.1186/s13046-018-0757-8 (PMC5902951; doi:10.1186/s13046-018-0757-8)
Supplement: Supplementary file 1 — Table S1. The groups of CRC patient population used in different figures. (DOC 29 kb) [file 13046_2018_757_MOESM1_ESM.doc]

**Additional file 1: Table S1. The groups of CRC patient population used in different figures.**

| Group | Number | Samples | Experiment | Figures |
| --- | --- | --- | --- | --- |
| 1 | 31 | Paired fresh tissues | miR-760 PCR | 1b, 1c, 1e |
| 2 | 45 | Paired fresh tissues | miR-760 PCR | 1b, 1c, 1e |
|  |  | Fixedcancer tissues | BATF3 IHC | 7a, 7b, 7c |
|  |  | 20 paired fresh/fixed tissues | BATF3 IHC and PCR | 7d, 7e |
| 3 | 35 | Fixedcancer tissues | BATF3 IHC | 7f |
