# Supplementary material for: MiR-760 suppresses human colorectal cancer growth by targeting BATF3/AP-1/cyclinD1 signaling
Source: J Exp Clin Cancer Res. 2018 Apr 16;37:83. doi: 10.1186/s13046-018-0757-8 (PMC5902951; doi:10.1186/s13046-018-0757-8)

**Additional file 2: Figure** **S4. MiR-760 inhibited human BATF3/c-Jun and downstream cyclin D1 in CRC cells. a.** Expression of c-Jun and BATF3 in SW620 (left) and HCT116 cells (right) transfected with the indicated miRNAs detected by Western Blot. **b.** Expression of c-Jun in SW620 (left) and HCT116 cells (right) transfected with the indicated miRNAs detected by real-time PCR. **c**. Real-time PCR analysis of cyclin D1, p21, and p27 mRNA expression in human CRC cell lines SW620, DLD1, HCT116, and colorectal mucosa cell line FHC.


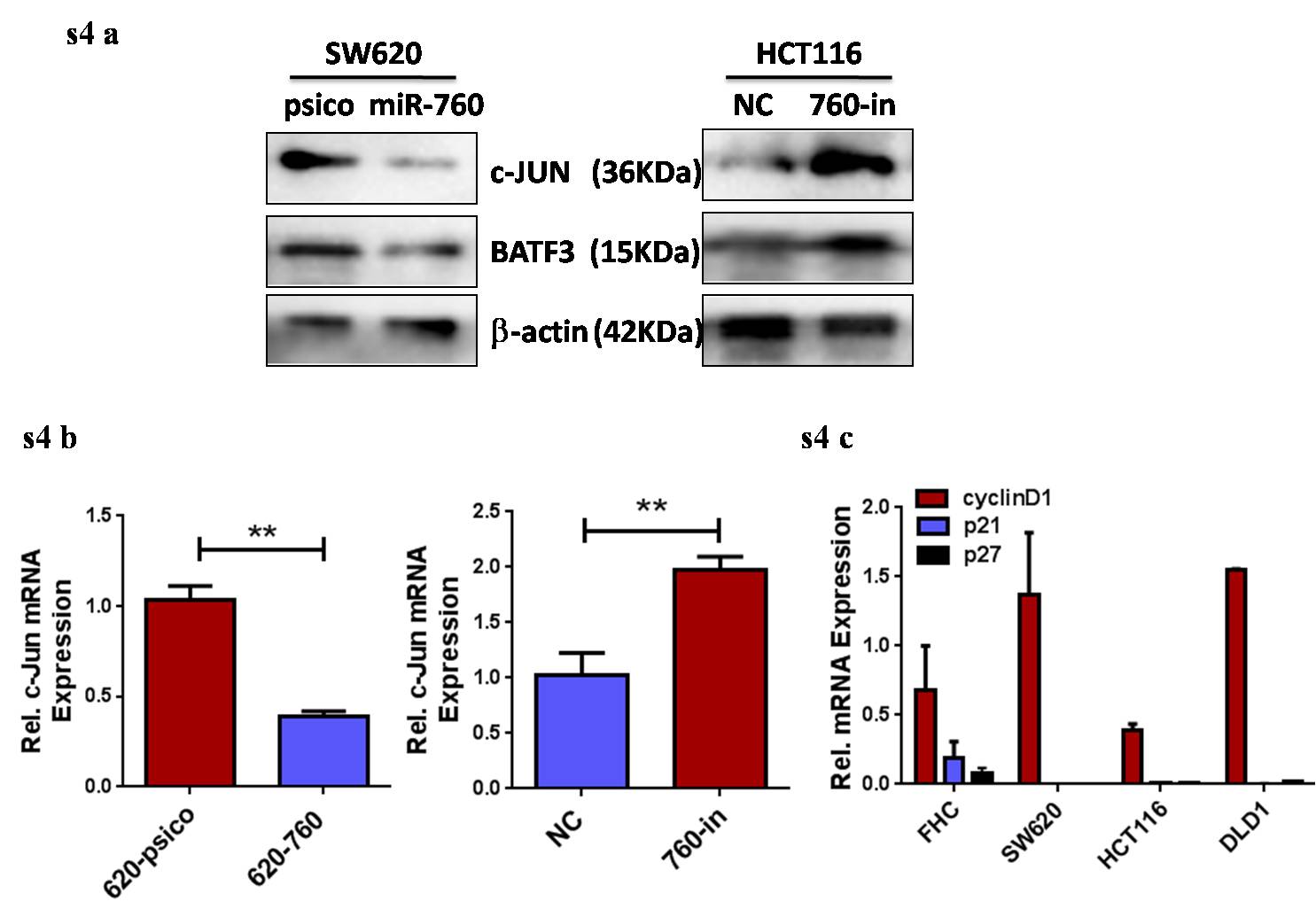

Supplement: Supplementary file 2 — Figure S4. MiR-760 inhibited human BATF3/c-Jun and downstream cyclin D1 in CRC cells. (DOCX 109 kb) [file 13046_2018_757_MOESM2_ESM.docx]
